# Gamma Glutamyl Transferase and Uric Acid Levels Can Be Associated with the Prognosis of Patients in the Pediatric Intensive Care Unit

**DOI:** 10.3390/children5110147

**Published:** 2018-10-30

**Authors:** Fatih Aygun, Ruhsar Kirkoc, Deniz Aygun, Halit Cam

**Affiliations:** 1 Department of Pediatric Intensive Care Unit, Istanbul University Cerrahpasa Medical Faculty, Fatih, 34098 Istanbul, Turkey; hacam@istanbul.edu.tr; 2 Department of Pediatrics, Okmeydanı Research and Training Hospital, 34384 Istanbul, Turkey; ruhsarkirkoc@hotmail.com; 3 Department of Infections Disease, Istanbul University Cerrahpasa Medical Faculty, 34098 Istanbul, Turkey; fdenizaygun@gmail.com

**Keywords:** gamma glutamyl transferase, uric acid, critically ill children, mortality

## Abstract

*Introduction*: Gamma glutamyl transferase (GGT) and uric acid (UA) are reported to be predictive markers in various disorders. It has been reported that these biomarkers can be used to indicate increased risk of mortality in critically ill patients. Herein, we aimed to evaluate the effects of the initial serum GGT and UA levels on the outcomes of patients in the pediatric intensive care unit (PICU) and to investigate if these biomarkers can be used to predict pediatric mortality. *Materials and Methods*: The relationship between the initial GGT and UA levels and invasive mechanical ventilation (IMV) and noninvasive mechanical ventilation (NIV) support, inotropic drug need, acute renal kidney injury (AKI), continuous renal replacement therapy (CRRT), presence of sepsis, mortality, and hospitalization period were investigated retrospectively. *Results*: In all, 236 patients (117 males and 119 females) were included in the study. The age distribution of the patients was 1–12 years. There was a statistically significant relationship between GGT levels in the first biochemical analysis performed during admission and inotropic drug use, AKI, duration of hospitalization in intensive care unit, and sepsis. There was a statistically significant relationship between initial UA levels and inotropic drug use, AKI, CCRT, and sepsis. *Conclusion*: We suggest that initial GGT and UA levels during admission could be used to predict the outcomes of patients in PICU.

## 1. Introduction

Gamma glutamyl transferase (GGT) is a plasma membrane enzyme that is commonly used in clinical practice as a marker for liver function. GGT can also be used for diagnosis of other disorders like myocardial infarction and neurological, neuromuscular, lung, and pancreatic disorders. Thus, the enzyme is present in extra-hepatic tissues including lymphocytes, fibroblasts, choroid plexus, epididymis, kidney, and lungs [1,2,3]. GGT catalyzes the hydrolysis of extracellular glutathione and is essential for antioxidant defense. Thus, it is suggested that GGT may be a predictor for diseases involving oxidative stress and inflammatory reactions [4,5,6].

Uric acid (UA), which is an end product of purine (adenosine and guanosine) catabolism is also considered as an antioxidant like GGT [7]. Elevated levels of UA have been shown to be associated with increased cellular damage and mortality of critically ill pediatric patients independent of the underlying disease [8].

Recently GGT and UA were reported as predictive markers in various disorders, such as acute myocardial infarction, cirrhosis, and sepsis, and it has been reported that these biomarkers can be used for indicating an increased risk of mortality in critically ill patients [1,3,8].

GGT and UA levels are not routinely analyzed in many pediatric intensive care units (PICUs) at admission and there are not enough studies that investigate the relationship between these biochemical markers and the prognosis of patients in PICU. Here, we aimed to evaluate the initial serum GGT and UA levels and the outcomes of patients in the PICU and to investigate if these biomarkers can be used to predict pediatric mortality like the Pediatric Risk of Mortality (PRISM) and Pediatric Logistic Organ Dysfunction (PELOD) scoring methods.

## 2. Materials and Methods

### 2.1. Study Design

The data of all patients admitted to PICU from October 2016 to September 2017 were reviewed retrospectively. Ethical approval for this study was obtained from the Local Ethics Committee (21.11.2017-763). The informed consent form for PICU treatment was obtained from patients’ parents on admission.

We have a tertiary, multidisciplinary PICU located in a training and research hospital. Our PICU provides healthcare for children aged between one month and eighteen years. It has twelve beds, eleven ventilators, and two isolation rooms. Our unit employs a pediatric intensive care specialist, two assistants, and 27 nurses.

### 2.2. Patient Population and Data Collection

As GGT levels vary according to age group, patients younger than 1 year and older than 12 years were not included in the study. Patients with liver disease, kidney disease, cyanotic congenital heart and metabolic disease, hematologic or oncologic disorders, or diabetes, or those who had undergone any surgical procedure were excluded from the study. In addition, patients who were discharged or died in less than 24 h, and patients with a deficiency in either GGT or UA at admission were not included.

The demographic, prognostic, and laboratory findings of patients were collected. The relationship between the initial GGT and UA levels and invasive mechanical ventilation (IMV) and noninvasive mechanical ventilation (NIV) support, inotropic drug need, acute renal kidney injury (AKI), continuous renal replacement therapy (CRRT), presence of sepsis, mortality, and hospitalization period were evaluated.

### 2.3. Laboratory Analysis

The initial biochemical markers, GGT, and UA test results were recorded. For assessment of biochemistry, peripheral blood was collected into vacutainer tubes and analyzed by the same machines (Beckman Coulter for biochemistry). GGT and UA were measured in fresh serum samples. Activity of GGT was expressed as U/L.

In the study by Heiduk et al., the upper limit of GGT was found to be 42.6 IU in the 1–17 years’ age group [9]. In another recent study, this limit was found to be 45 IU in children [10]. The highest GGT values in both studies were found in adolescent children. Therefore, while we were planning our study, we considered the upper limit of the GGT to be 40 IU. We compared patients with GGT < 40 IU and GGT > 40 IU. The laboratory reference values of UA were 3.5–7.2 mg/dL in our hospital.

### 2.4. Statistical Analysis

The SPSS program (version 20.0, IBM Corporation, SPSS Inc., Chicago, IL, USA) was used for statistical analyses. Numerical data were expressed as mean ± standard deviation, while categorical data were expressed as frequency (*n*) and percentage (%). Pearson’s chi-square and analysis of variance (ANOVA) tests were used for the comparison of categorical data between groups. The receiver operating characteristic (ROC) curve was used to assess the performance of GGT and UA and their relationship with the prognostic factors. Univariate binary logistic regression models were conducted to calculate the odds ratio (OR) with a 95% confidence interval (CI) for GGT > 40 IU and UA > 7 mg/dL. *p* < 0.05 was considered to be statistically significant.

## 3. Results

**Figure 1:**
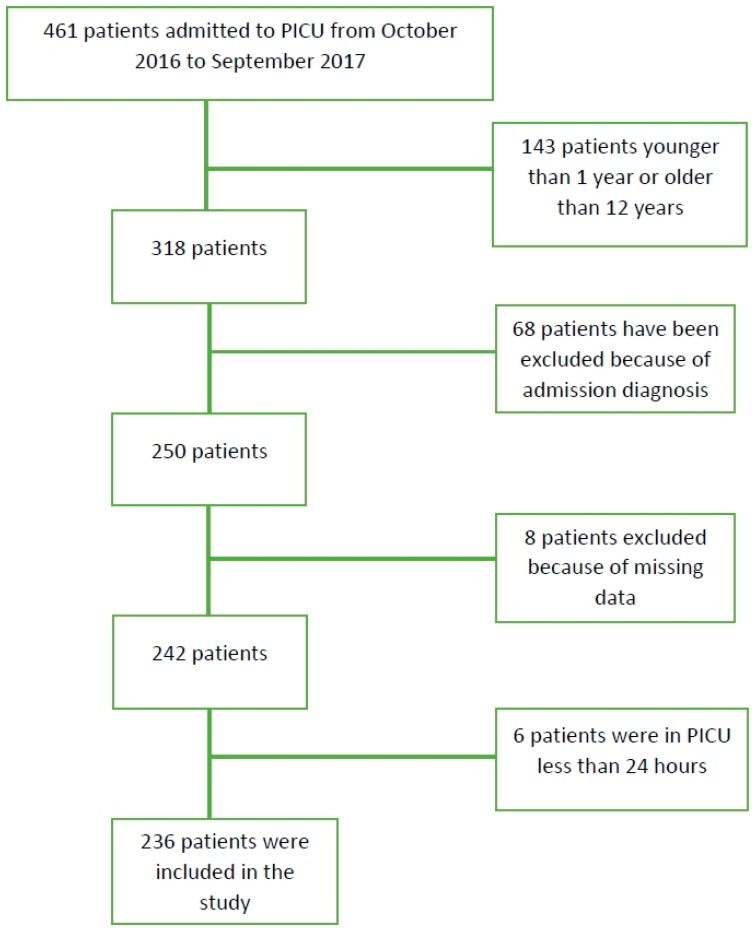
Cohort flow.

A total of 461 patients were evaluated. Two hundred and twenty-five patients were not included in the study because they did not meet the study criteria; 14 patients had a history of renal disease, 12 patients had diabetes mellitus, 12 patients had congenital metabolic disease, 15 patients had hematologic or oncologic disorders, 8 patients had liver disease or cholestasis, and 7 patients had cyanotic congenital disease. Six patients were excluded because they were either discharged from the intensive care unit or died in less than 24 h. In addition, eight patients were excluded because they did not receive initial GGT and UA analysis at admission. One hundred and forty-three patients younger than 1 year of age and older than 12 years of age were not included in the study. The remaining 236 patients were suitable for the study (Figure 1). The demographic characteristics of the patients are shown in Table 1.

One hundred and seventeen (49.6%) patients were male and 119 (50.4%) were female. Age distributions ranged from 1–12 years of age with a mean of 5.75 ± 5.16 years. The most frequent disease diagnoses were respiratory disorders (75 patients; 31.8%), neurological diseases (42 patients; 17.8%), sepsis (38 patients; 16.1%), and intoxication (37; 15.7%). The mean value for the duration of stay in PICU was 7.72 ± 10.69 days while the mean PRISM-III score was 14.24 ± 13.46.

IMV was used in 58 (24.6%) patients and NIV was used in 90 (38.1%) patients. AKI developed in 54 (22.9%) patients during PICU hospitalization and 24 (10.2%) of these patients underwent CRRT. Fifty (21.2%) patients were treated with inotropic drugs, extracorporeal membrane oxygenation (ECMO) was performed in 4 (1.7%) patients, and 7 of the patients (3.0%) died during PICU hospitalization. Table 2 shows the statistically significant relationships between GGT levels in the first biochemical analysis performed during admission and IMV and NIV support, inotropic drug use, AKI, duration of hospitalization in intensive care unit, and sepsis with p values of 0.001, 0.014, 0.015, 0.022, 0.001, and 0.000 respectively. There were statistically significant relationships between UA levels and IMV support, inotropic drug use, AKI, CCRT and sepsis. The *p* values were 0.001, 0.000, 0.000, 0.001, and 0.017, respectively.

Analysis of ROC curves showed that GGT levels were associated with NIV (area under the curve (AUC):0.651), IMV (AUC:609), AKI (AUC:0.665), CRRT (AUC:634), sepsis (AUC:0.609), inotropic drug use (AUC:0.642), and LOS (AUC:707) (Table 3). In addition, analysis of ROC curves showed that UA levels were associated with AKI (AUC:0.735), CRRT (AUC:662), sepsis (AUC:0.668), and inotropic drug use (AUC:0.647) (Table 4).

The ORs and relationships between prognostic factors and GGT > 40 IU were calculated using logistic regression models. ORs were 3.753 (CI, 1.325–10.631) for acute kidney injury, 4.059 (CI, 1.193–13.808) for inotropic drug use, 2.685 (CI, 1.069–6.742) for length of stay (>7 d), and 2.345 (CI, 0.897–5.456) for sepsis (Table 5 and Table 6).

The ORs and relationships between prognostic factors and UA > 7 mg/dL were also calculated. ORs were 10.921 (CI, 3.369–35.396) for acute kidney injury, 3.518 (CI, 1.615–10.301) for inotropic drug use, and 4.348 (CI, 1.356–13.938) for sepsis (Table 5 and Table 6).

## 4. Discussion

In this study, we evaluated the relationship between GGT and UA levels and possible risk factors. Higher GGT and UA values were associated with increased AKI, inotropic drug usage, and sepsis in critically ill children. The ROC analyses showed that GGT and UA were associated with prognostic factors. Therefore, we thought that these markers could be used in predicting prognoses, similar to mortality scores.

Many scoring systems such as PELOD and PRISM have been developed according to the laboratory and clinical symptoms of patients to determine mortality risk and disease severity in PICUs. However, many other factors can affect the mortality of patients in intensive care units (ICUs). In recent studies, the relationships between GGT and UA levels and the prognosis of critically ill patients have been investigated separately in adults and these biomarkers have been suggested as prognostic factors in mortality [1,4,8]. In this study, GGT and UA were not directly associated with mortality.

It is well-documented that IMV is an independent risk factor in the prognosis of children in PICU [11,12,13]. In this study, GGT was significantly higher in patients with IMV use, and the ROC curves showed that the GGT score covered an AUC of 0.609 for a cutoff value of 30.6 IU. In addition, GGT level was associated with NIV (AUC: 0.651).

The prolonged duration of hospitalization in PICUs and the use of inotropic drugs have also been reported to be associated with a poor prognosis [8]. In this study, higher GGT values were associated with a prolonged PICU stay (Table 2). The ROC curves showed that the GGT score covered an AUC of 0.707 for a cutoff value of 38.5 IU for prolonged PICU stay.

There was a statistically significant relationship between GGT levels and inotropic drug use, AKI, duration of hospitalization in intensive care unit and sepsis. AKI is another independent risk factor in the prognosis of critically ill patients that is associated with increased mortality, prolonged hospitalization and costs [14]. In our study, ROC curves showed that AKI had a high AUC (0.665). In addition, the logistic regression analysis showed that GGT > 40 IU increased AKI 3.753 fold. It is known that GGT levels increase as a consequence of severe cell damage due to various causes.

Increased GGT levels are associated with poor outcomes in sepsis and cardiovascular diseases in adults [1,2,3,4,5,6]. Sepsis is also universally accepted as a risk factor in ICUs [15]. GGT is considered as a predictor for diseases involving oxidative stress and inflammatory reactions. The enzyme antioxidant system is the first line of defense against the damage produced by reactive substances, and the inflammation increases those enzymes. In GGT, this enzyme membrane helps to counterbalance the oxidative process [16]. In addition, GGT is part of the gamma glutamyl cycle which plays a key role in maintaining reduced glutathione (GSH) homeostasis. Serum GGT restores baseline levels of GSH. Therefore, higher GGT activity due to oxidative stress contributes to increases in the GSH [17,18]. In this study, we have shown that sepsis, which is one of the diseases that most increases oxidative stress, is associated with GGT.

Therefore, we can state that increased GGT levels are also associated with prognoses in children but we could not make any comparison because of the lack of literature regarding GGT levels in PICUs. Although, there are a few studies in neonates regarding the reference range of GGT [8,19,20] there is no study yet that investigates the importance of GGT in the prognosis of critically ill children. Our study showed that GGT levels were associated with the length of stay in PICUs (>7 d) (AUC:0.707). In addition, GGT levels were associated with prognostic factors. This result was also confirmed by the ROC analysis and logistic regression models.

There were statistically significant relationships between UA levels and IMV support, inotropic drug use, AKI, CRRT and sepsis. In our study, ROC curves showed that AKI had a higher AUC than the others (0.735). Hooman et al. reported no relationship between elevated UA levels and usage of IMV [8]. The main difference between that study and ours are the exclusion criteria and we believe that it is necessary to exclude diseases that could increase the UA levels.

Similar to our report, Aminiahidashti reported no relationship between serum UA level and the mortality rate of patients, although serum UA levels were higher in patients who underwent IMV [21]. In another report, increased UA levels were associated with prolonged hospitalization time and also associated with increased duration of mechanical support [22]. In our study, this relationship was not seen between UA and IMV in the ROC and logistic regression analysis. The UA showed an AUC of 0.568 for IMV and an AUC of 0.486 for length of PICU stay (>7 d). There are only a few reports in the literature about the relationship of increased UA levels with the prognosis of children in PICU [8,23]. Our study showed that UA levels were associated with AKI (AUC: 0.735), sepsis (AUC: 0.668) and inotropic drug use (AUC:0.647). The logistic regression analysis showed that AKI increased mortality 10.921 fold.

In the present study, we also evaluated the association with mortality. However, we found no statistically significant difference in terms of mortality and all of the biochemical markers. Hsai et al. reported that hyperuricemia is associated with increased risk of mortality in children and adolescents, rather than the severity of the underlying disease. In the same study, mortality was higher in patients who had cardiovascular and renal diseases along with hyperuricemia [24]. Although we could not demonstrate any statistical correlation between GGT and UA levels and mortality, we believe that they may be indirectly associated with mortality.

There are some limitations to our study. Our study is retrospective and single-centered. In addition, serial measurement of two biomarkers was missing. Concomitant medication, sedation, fever, nosocomial infections, and other complications were not evaluated. However, the fact that such a study has not been done before in a pediatric age group makes our study valuable. The other strength of this study is that a relatively large number of patients were included. In addition, there is a need for prospective and multicenter studies involving more patients.

## 5. Conclusions

GGT and UA, are cost effective, useful, and easily accessible tests for prognosis in critically ill children. This study demonstrated that admission GGT and UA values are closely associated with sepsis, AKI, and inotropic drug usage in the PICU.

In conclusion, we suggest that initial GGT and UA levels during admission can be used to predict the outcome of patients in PICUs.

## Figures and Tables

**Table 1:** Demographic characteristics of patients.

Number of Patients	(*n* = 236)
**Gender**	*n* (%)
Male	117 (49.6%)
Female	119 (50.4%)
**Reasons for Hospitalization**	
Respiratory System Disease	75 (31.8%)
Neurologic Disease	42 (17.8%)
Sepsis	38 (16.1%)
Intoxication	37 (15.7%)
Cardiovasular Disease	10 (4.2%)
Trauma	14 (5.9%)
Other	20 (8.5%)
**Age (years)**	Mean ± S.D.
1–12	(5.75 ± 5.16)
**Prognostic factors**	*n* (%)
Acute Kidney Injury	54 (22.9%)
Inotropic medication	50 (21.2%)
CRRT ^1^	24 (10.2%)
Mechanical Ventilation	58 (24.6%)
NIV ^3^	90 (38.1%)
ECMO ^4^	4 (1.7%)
Death	7 (3.0%)
Duration of Stay in PICU ^2^ (d)	7.72 ± 10.69
PRISM ^5^	14.24 ± 13.46

^1^ continuous renal replacement therapy; ^2^ patients in pediatric intensive care unit (PICU); ^3^ noninvasive mechanical ventilation; ^4^ extracorporeal membrane oxygenation. ^5^ pediatric risk of mortality.

**Table 2:** Evaluation of prognostic factors according to gamma glutamyl transferase (GGT) and uric acid (UA) levels: 1. Gamma glutamyl transferase. 2. Uric acid. 3. Noninvasive Mechanical Ventilation. 4. Continuous Renal Replacement Treatment.

	GGT (U/L) ^1^ Mean ± SD	UA (mg/dl) ^2^ Mean ± SD	*p* Value
**Invasive Mechanical Ventilation**			
Yes	64.29 ± 88.21	5.02 ± 3.25	(1) 0.001
No	38.37 ± 57.50	4.03 ± 2.11	(2) 0.001
**NIV ^3^**			
Yes	53.18 ± 70.14	4.26 ± 2.58	(1) 0.041
No	38.74 ± 66.69	4.36 ± 2.51	(2) 0.712
**Inotropic drug usage**			
Yes	60.89 ± 80.93	5.44 ± 3.15	(1) 0.015
No	40.87 ± 63.41	3.96 ± 2.19	(2) 0.000
**Mortality**			
Yes	75.62 ± 89.87	5.39 ± 3.20	(1) 0.111
No	44.71 ± 67.63	4.28 ± 2.51	(2) 0.123
**Acute Kıdney Injury**			
Yes	60.66 ± 78.44	6.33 ± 3.34	(1) 0.022
No	41.39 ± 64.91	3.72 ± 1.87	(2) 0.000
**Length of PICU stay**			
<7 d	37.03 ± 55.06	4.23 ± 2.24	(1) 0.001
7 d and more	60.98 ± 85.38	4.47 ± 2.98	(2) 0.387
**CRRT ^4^**			
Yes	56.21 ± 92.10	5.81 ± 2.84	(1) 0.395
No	44.91 ± 66.37	4.19 ± 2.48	(2) 0.001
**Sepsis**			
Yes	53.62 ± 75.32	4.51 ± 2.70	(1) 0.000
No	24.87 ± 39.06	3.81 ± 1.95	(2) 0.017

^1^ gamma glutamyl transferase; ^2^ uric acid; ^3^ noninvasive mechanical ventilation; ^4^ continuous renal replacement treatment.

**Table 3:** Analysis of gamma glutamyl transferase (GGT) and prognostic factors by receiver operating characteristic (ROC) analysis in PICU patients.

Parameter	Area Under Curve	S.E.	*p* Value	95% C.I.		Cut-Off Value	Sensitivity	Specificity
				Lower Bound	Upper Bound			
NIV	0.651	0.036	0.000	0.580	0.721	32.5	71.1%	53.8%
IMV	0.609	0.042	0.013	0.526	0.692	30.6	69.0%	48.3%
AKI	0.665	0.043	0.000	0.580	0.750	29.2	70.4%	52.3%
CRRT	0.634	0.063	0.032	0.511	0.757	34.5	66.7%	60.8%
Sepsis	0.609	0.050	0.039	0.512	0.706	33.5	74.3%	51.7%
Inotropic drug	0.642	0.044	0.002	0.557	0.728	34.5	70.0%	58.1%
LOS (>7 days)	0.707	0.036	0.000	0.636	0.778	38.5	72.4%	51.5%

S.E.: standard error, C.I.: confidence interval, NIV: noninvasive mechanical ventilation, IMV: invasive mechanical ventilation, AKI: acute kidney injury, CRRT: continuous renal replacement treatment, LOS: length of stay.

**Table 4:** Analysis of uric acid and prognostic factors by ROC analysis in PICU patients.

Parameter	Area Under Curve	S.E.	*p* Value	95% C.I.		Cut-Off Value	Sensitivity	Specificity
				Lower Bound	Upper Bound			
NIV	0.457	0.040	0.274	0.377	0.536	3.48	48.8%	46.4%
IMV	0.568	0.048	0.122	0.475	0.662	3.59	59.6%	55.3%
AKI	0.735	0.044	0.000	0.649	0.820	4.19	73.1%	70.3%
CRRT	0.662	0.065	0.011	0.534	0.790	3.49	69.7%	63.7%
Sepsis	0.668	0.055	0.002	0.560	0.776	4.14	68.6%	65.1%
Inotropic drug	0.647	0.048	0.002	0.554	0.740	4.19	62.5%	66.5%
LOS (>7 days)	0.486	0.047	0.738	0.6394	0.578	3.21	59.1%	55.6%

S.E.: standard error, C.I.: confidence interval, NIV: noninvasive mechanical ventilation, IMV: invasive mechanical ventilation, AKI: acute kidney injury, CRRT: continuous renal replacement treatment, LOS: length of stay.

**Table 5:** Risk factors of patients with GGT > 40 IU (binary logistic regression models).

RISK	*p* Values	Odds Ratio	95% Confidence Interval
Mechanical Ventilation	0.392	1.526	0.580–4.017
Noninvasive Mechanical Ventilation	0.752	1.128	0.499–2.078
Inotropic drug usage	0.025	4.059	1.193–13.808
Acute Kidney Injury	0.013	3.753	1.325–10.631
Continuous Renal Replacement Therapy	0.701	1.268	0.376–4.285
Length of Stay (>7 days)	0.036	2.685	1.069–6.742
Mortality	0.119	1.868	0.851–4.104
Sepsis	0.044	2.345	0.897–5.456

**Table 6:** Risk factors of patients with UA > 7 mg/dL (binary logistic regression models).

RISK	*p* Values	Odds Ratio	95% Confidence Interval
Mechanical Ventilation	0.233	2.075	0.625–6.885
Noninvasive Mechanical Ventilation	0.462	1.458	0.399–3.038
Inotropic drug usage	0.049	3.518	1.615–10.301
Acute Kidney Injury	0.000	10.921	3.369–35.396
Continuous Renal Replacement Therapy	0.797	1.175	0.345–4.005
Length of Stay (>7 days)	0.666	1.311	0.384–4.471
Mortality	0.518	0.705	0.245–2.033
Sepsis	0.013	4.348	1.356–13.938
